# Supplementary material for: Meta‐analysis of dichotomous and ordinal tests with an imperfect gold standard
Source: Res Synth Methods. 2022 Jun 25;13(5):595–611. doi: 10.1002/jrsm.1567 (PMC9541315; doi:10.1002/jrsm.1567)
Supplement: Supplementary file 1 — Appendix S1 Supporting Information [file JRSM-13-595-s001.pdf]

Appendices

1 Technical model specification

The Bayesian MVP-LC model we proposed assumes that there are S Studies each assessing $T \geq 2$ tests with N_s individuals in each study, $s \in \{1, \ldots, S\}$. We assume that all studies report data on all categories of each test, and each test, $t \in \{1, \ldots, T\}$, has K_t categories ($K_t - 1$ cutpoints).

1.1 Within-study model

Within each study s, each individual $n \in \{1, ..., N_s\}$ has a vector of observed test responses, $\mathbf{y}_{s,n}$, equal to

$$\mathbf{y}_{s,n} = \{y_{s,n,1}, \ldots, y_{s,n,T}\}$$

Assume that each individual has latent disease status $d = d_{s,n} \in \{0 = \text{non-diseased}, 1 = \text{diseased}\}$. Conditional on the disease status of each individual, $d = d_{s,n} \in \{0, 1\}$, we augment the observed data with normally distributed latent variables $\mathbf{Z}_{s,n}$,

$$\mathbf{Z}_{s,n} \sim \text{MVN}\left(\boldsymbol{\nu}_s^{[d]}, \boldsymbol{\Psi}_s^{[d]}\right), \tag{1.1}$$

Where,

$$\mathbf{Z}_{s,n} = \begin{pmatrix} Z_{s,n,1} \\ \vdots \\ Z_{s,n,T} \end{pmatrix}, \boldsymbol{\nu}_s^{[d]} = \begin{pmatrix} \nu_{s,1}^{[d]} \\ \vdots \\ \nu_{s,T}^{[d]} \end{pmatrix}, \boldsymbol{\Psi}_s^{[d]} = \begin{pmatrix} (\tau_{s,1}^{[d]})^2 & \cdots & \dot{\epsilon}_{s,1,T}^{[d]} \cdot \tau_{s,1}^{[d]} \cdot \tau_{s,T}^{[d]} \\ \vdots & \ddots & \vdots \\ \dot{\epsilon}_{s,T,1}^{[d]} \cdot \tau_{s,T}^{[d]} \cdot \tau_{s,1}^{[d]} & \cdots & (\tau_{s,T}^{[d]})^2 \end{pmatrix}$$

We assume that the study-specific location parameters can be modelled by the unconstrained parameters $\nu_{s,t}^{[1]} \in \mathbb{R}$ and $\nu_{s,t}^{[0]} \in \mathbb{R}$ for the latent diseased and non-diseased populations, respectively. For the variance-covariance matrices $\boldsymbol{\Psi}_s^{[d]}$, for identifiability we need to set some restrictions[1,2]. In this paper, we set $\tau_{s,t}^{[d]} = 1, \forall s \in \{1, \ldots, S\}, \forall t \in \{1, \ldots, T\}$, so that each $\boldsymbol{\Psi}_s^{[d]}$ is a correlation matrix. This has the same form to the models proposed in Xu et al 2009[3] and Xu et al 2013[4]. Note that the correlations, $\dot{\epsilon}_{s,t,t'}^{[d]}$, represent the pairwise correlation between the latent variables for tests t and t' in study s, conditional on the disease status d. $\dot{\epsilon}_{s,t,t'}^{[d]}$ is referred to as the *polychoric* correlation[2,5]. If it is considered reasonable in a particular setting to assume that tests are conditionally independent given disease status, the correlations can be set to zero, so that $\boldsymbol{\Psi}_s^{[d]} = \text{diag}(1, \ldots, 1)$. For dichotomous test t, the observed test results of each individual are given by,

$$y_{s,n,t} = \begin{cases} 0 & \text{if } Z_{s,n,t} \leq 0 \\ 1 & \text{if } Z_{s,n,t} > 0 \end{cases} \tag{1.2}$$

For ordinal tests, the observed test results of each individual for test t are given by,

$$y_{s,n,t} = \begin{cases} 1 & \text{if } Z_{s,n,t} \leq C_{1,s,t}^{[d]} \\ 2 & \text{if } C_{1,s,t}^{[d]} < Z_{s,n,t} \leq C_{2,s,t}^{[d]} \\ \vdots & \\ K_t - 1 & \text{if } C_{K_t-2,s,t}^{[d]} < Z_{s,n,t} \leq C_{K_t-1,s,t}^{[d]} \\ K_t & \text{if } Z_{s,n,t} > C_{K_t-1,s,t}^{[d]} \end{cases} \tag{1.3}$$

Where $C_{k,s,t}^{[d]} < C_{k+1,s,t}^{[d]}$ for $k \in \{1, \ldots, K_t - 2\}$ are the latent cutpoint parameters. Conditional on the true disease status of each individual, $d = d_{s,n} \in \{0, 1\}$, the probability of observing the test response vector $\mathbf{y}_{s,n}$ is given by,

$$P\left(\mathbf{y}_{s,n} | d = d_{s,n}, \boldsymbol{\nu}_s^{[d]}, \boldsymbol{\Psi}_s^{[d]}, \mathbf{C}_{k,s}^{[d]}\right) = \int_{I_{s,n,1}^{[d]}} \cdots \int_{I_{s,n,T}^{[d]}} \Phi_T\left(k | \boldsymbol{\nu}_s^{[d]}, \boldsymbol{\Psi}_s^{[d]}\right) dk \tag{1.4}$$

where $\Phi_T\left(\ \cdot\ |\boldsymbol{\nu}_s^{[d]}, \boldsymbol{\Psi}_s^{[d]}\right)$ denotes the cumulative distribution function of a multivariate normal distribution with dimension equal to the number of tests in each study, T , with mean vector $\boldsymbol{\nu}_s^{[d]}$ and variance-covariance matrix $\boldsymbol{\Psi}_s^{[d]}$. For dichotomous tests, the intervals $I_{s,n,t}$ are defined by,

$$I_{s,n,t}^{[d]} = \begin{cases} (-\infty,\ 0] & \text{if } y_{s,n,t} = 0 \\ (0,\ \infty) & \text{if } y_{s,n,t} = 1 \end{cases} \tag{1.5}$$

This corresponds to a binary latent class multivariate probit model with probability density function, $\pi(\cdot)$, is given by,

$$\pi(k \mid \nu_{s,t}^{[d]}) = \begin{cases} 1 - \Phi\left(\nu_{s,t}^{[d]}\right) & \text{if } k = 0 \\ \Phi\left(\nu_{s,t}^{[d]}\right) & \text{if } k = 1 \end{cases} \tag{1.6}$$

Where $\Phi(\cdot)$ denotes the cumulative density function of the standard normal distribution. The measures of test accuracy for each study are given by,

$$\begin{aligned} Se_{s,t} &= \Phi(\nu_{s,t}^{[1]}) \\ Sp_{s,t} &= 1 - \Phi(\nu_{s,t}^{[0]}) \end{aligned} \tag{1.7}$$

For ordinal tests, the intervals, $I_{s,n,t}^{[d]}$, are defined by,

$$I_{s,n,t}^{[d]} = \begin{cases} (-\infty,\ C_{1,s,t}^{[d]}] & \text{if } y_{s,n,t} = 1 \\ (C_{1,s,t}^{[d]},\ C_{2,s,t}^{[d]}] & \text{if } y_{s,n,t} = 2 \\ \vdots & \\ (C_{K_t-2,s,t}^{[d]},\ C_{K_t-1,s,t}^{[d]}] & \text{if } y_{s,n,t} = K_t - 1 \\ (C_{K_t-1,s,t}^{[d]},\ \infty) & \text{if } y_{s,n,t} = K_t \end{cases} \tag{1.8}$$

This corresponds to an ordered latent class multivariate probit model[2], with probability density function for test t given by,

$$\pi(k \mid \nu_{s,t}^{[d]}, \mathbf{C_{s,t}^{[d]}}) = \begin{cases} \Phi(C_{1,s,t}^{[d]} - \nu_{s,t}^{[d]}) & \text{if } k = 1, \\ \Phi(C_{k,s,t}^{[d]} - \nu_{s,t}^{[d]}) - \Phi(C_{k-1,s,t}^{[d]} - \nu_{s,t}^{[d]}) & \text{if } 1 < k < K_t, \\ 1 - \Phi(C_{K_t-1,s,t}^{[d]} - \nu_{s,t}^{[d]}) & \text{if } k = K_t. \end{cases} \tag{1.9}$$

Therefore, for a test which has decreasing sensitivity and increasing specificity with increasing cutpoint, the measures of test accuracy for test t at a cutpoint of k in study s are given by,

$$\begin{aligned} Se_{s,t,k} &= 1 - \Phi(\nu_{s,t}^{[1]} - C_{k,s,t}^{[1]}) \\ Sp_{s,t,k} &= \Phi(\nu_{s,t}^{[0]} - C_{k,s,t}^{[0]}) \end{aligned} \tag{1.10}$$

The likelihood contribution from each study $s \in \{1, ..., S\}$ is given by a latent class model with two classes, where one component corresponds to the diseased group and the other to the non-diseased group. Using equation (1.4), we can write the likelihood function for each study as the sum of the log probability terms for each individual study,

$$\log L\left(\boldsymbol{\theta}|\mathbf{y}_s\right) = \sum_{n_s=1}^{N_s} \log\left[\ p_s \cdot P\left(\mathbf{y}_{n,s}|d_n = 1, \boldsymbol{\nu}_s^{[1]}, \boldsymbol{\Psi}_s^{[1]}, \mathbf{C}_{k,s}^{[1]}\right)\ +\ (1-p_s) \cdot P\left(\mathbf{y}_{n,s}|d_n = 0, \boldsymbol{\nu}_s^{[0]}, \boldsymbol{\Psi}_s^{[0]}, \mathbf{C}_{k,s}^{[0]}\right)\ \right]$$

Where $p_s,\ s \in \{1, \ldots, S\}$ denotes the disease prevalence in each study and $\boldsymbol{\theta}$ denotes the vector of model parameters.

Using the augmented latent variables, $Z_{s,n,t}$, we can write this as,

$$\log L\left(\boldsymbol{\theta}|\mathbf{y}_s\right) = \sum_{n=1}^{N_s} d_n \cdot \log(p_s) + \sum_{n=1}^{N_s} d_n \cdot \log[\ \Phi_T\left(\mathbf{z}_{s,n}|\boldsymbol{\nu}_s^{[1]}, \boldsymbol{\Psi}_s^{[1]}\right)\ ] + \sum_{n=1}^{N_s}(1-d_n)\cdot\log(1-p_s) + \sum_{n=1}^{N_s}(1-d_n)\cdot\log[\ \Phi_T\left(\mathbf{z}_{s,n}|\boldsymbol{\nu}_s^{[0]}, \boldsymbol{\Psi}_s^{[0]}\right)\ ] + \sum_{n=1}^{N_s}[d_n \cdot \sum_{t=1}^{T}\log(z_{s,n,t}\in I_{s,n,t}^{[1]}) + (1-d_n)\cdot\sum_{t=1}^{T}\log(z_{s,n,t}\in I_{s,n,t}^{[0]})] \tag{1.11}$$

1.2 Between-study model

Recall that $\nu_{s,t}^{[d]}$ are the location parameters for study s, test t in latent population d. We define a vector $\boldsymbol{\nu}_{s,t} = (\nu_{s,t}^{[1]}, \nu_{s,t}^{[0]})'$ and assume a partial pooling, bivariate normal population model,

$$\pi(\boldsymbol{\nu}_{s,t} \mid \theta) = \text{MVN}(\boldsymbol{\mu}_t, \boldsymbol{\Sigma}_t), \tag{1.12}$$

Where $\boldsymbol{\mu}_t = (\mu_t^{[1]}, \mu_t^{[0]})'$ is a vector containing the mean parameters, and $\boldsymbol{\Sigma}_t = \begin{pmatrix} \left(\sigma_t^{[1]}\right)^2 & \rho_t \cdot \sigma_t^{[1]} \cdot \sigma_t^{[0]} \\ \rho_t \cdot \sigma_t^{[1]} \cdot \sigma_t^{[0]} & \left(\sigma_t^{[0]}\right)^2 \end{pmatrix}$ is a variance-covariance matrix, where $\sigma_t^{[1]}$ and $\sigma_t^{[0]}$ represent the between-study standard deviations for the sensitivities and specificities, respectively, and ρ_t represents the between-study correlation between sensitivities and specificities. We can set a given test, t', to be a perfect gold standard (100% sensitive and specific) by setting $\mu_{t'}^{[0]} = -5$ and $\mu_{t'}^{[1]} = 5$, which correspond to 100% specificity and sensitivity, respectively, and by using a complete pooling model (in other words, assuming zero between study heterogeneity i.e. $\sigma_{t'}^{[d]} = 0$).

1.2.1 Meta-regression

We can incorporate meta-regression covariates into the model. Let $\mathbf{X}_{1,t}\ldots\mathbf{X}_{M,t}$ be M vectors meta-regression covariates such that each $\mathbf{X}_{m,t} = (X_{m,1,t},\ldots,X_{m,S,t}) \in \mathbb{R}^S$. Let $\boldsymbol{\gamma}_{1,t}\ldots\boldsymbol{\gamma}_{M,t}$ be M vectors of meta-regression coefficients, such that each $\mathbf{fl}_{m,t} = (\gamma_{m,t}^{[1]}, \gamma_{m,t}^{[0]})' \in \mathbb{R}^2,\ m \in \{1\ldots M\}$. Then we write (1.12) as,

$$\pi(\boldsymbol{\nu}_{s,t} \mid \theta) = \text{MVN}(\mu_t + X_{1,s,t}\cdot\boldsymbol{\gamma}_{1,t} + \ldots + X_{M,s,t}\cdot\boldsymbol{\gamma}_{M,t}, \boldsymbol{\Sigma}_t), \tag{1.13}$$

For the disease prevalence in each study, we implement a *no pooling* (i.e., independent effects) model, which does not assume any latent interactions between the individual disease prevalence parameters,

$$\pi(p_1,\ldots,p_S) = \prod_{s=1}^{S}\pi(p_s) \tag{1.14}$$

1.2.2 Cutpoint model

The cutpoint parameters can be modelled using an induced Dirichlet model, an approach which has been proposed by Betancourt[6], which we describe in more detail in Appendix 1 **section ......** This model applies a Dirichlet model directly to the ordinal probabilities, by mapping the latent cut point parameters in each study $\{C_{1,s,t}^{[d]},\ldots,C_{K_t-1,s,t}^{[d]}\}$ to the simplex of ordinal probabilities $\{P_{1,s,t}^{[d]},\ldots,P_{K_t,s,t}^{[d]}\}$ using an injective (i.e. one-to-one) function. The probability density function for the induced Dirichlet model is given by,

$$\text{Induced-Dir}\left(\mathbf{C}_{s,t}^{[d]}, \mid \boldsymbol{\alpha}_{s,t}^{[d]}, \phi\right) = \text{Dir}\left(\mathbf{P}(\mathbf{C}_{s,t}^{[d]}, \phi) \mid \boldsymbol{\alpha}_{s,t}^{[d]}\right)\cdot\left|J\left(\mathbf{C}_{s,t}^{[d]}\right)\right|, \tag{1.15}$$

Where $\mathbf{C}_{s,t}^{[d]} = \left(C_{1,s,t}^{[d]}, \ldots, C_{K_t-1,s,t}^{[d]} \right)'$ is the vector of cutpoints for study s and test t, $\boldsymbol{\alpha}_{s,t}^{[d]} = \left(\alpha_{1,s,t}^{[d]}, \ldots, \alpha_{K_t,s,t}^{[d]} \right)'$ is the Dirichlet vector for study s and test t, $\mathbf{P}(\mathbf{C}_{s,t}^{[d]}, \phi)$ represents the induced ordinal probabilities in terms of the cutpoints $\mathbf{C}_{s,t}^{[d]}$ and an arbitrary anchor point ϕ, and $\left| J\left(\mathbf{C}_{s,t}^{[d]} \right) \right|$ is the determinant of the Jacobian matrix of partial derivatives (we need a Jacobian adjustment since we are directly modelling transformed parameters). We can use the induced Dirichlet model to directly specify a complete pooling model on the cutpoints by setting $\mathbf{C}_{s,t}^{[d]} = \mathbf{C}_t^{[d]}$ $\forall s$, and specifying $\boldsymbol{\alpha}_{s,t}^{[d]}$ $\forall s,\ t$ as constants. We can specify a partial pooling model on the cutpoints by setting $\boldsymbol{\alpha}_{s,t}^{[d]} = \boldsymbol{\alpha}_t^{[d]}$ $\forall s$ as parameters, so that,

$$\pi\left(\mathbf{C}_{s,t}^{[d]} | \theta \right) = \text{Induced-Dir}\left(\mathbf{C}_{s,t}^{[d]} | \boldsymbol{\alpha}_t^{[d]} \right) \cdot \pi\left(\boldsymbol{\alpha}_t^{[d]} \right) \tag{1.16}$$

In this case, we can obtain an 'average' vector of cutpoints, $\mathbf{C}_t^{[d]}$, by simulating repeatedly from (1.16) and averaging across draws.

<u>More detail</u>

The induced Dirichlet model allows us to move away from the abstract latent space in which the cutpoints are defined, and applies a Dirichlet model directly to the ordinal probabilities. We need to find an injective (i.e. one-to-one) function which maps the latent cut point parameters in each study $\{C_{1,s,t}^{[d]}, \ldots, C_{K_t-1,s,t}^{[d]}\}$ to the ordinal probabilities $\{P_{1,s,t}^{[d]}, \ldots, P_{K_t,s,t}^{[d]}\}$. Let $S^{[d]} = \sum_{k=1}^{K} P_{k,s,t}^{[d]} = 1$ and let $g : \mathbb{R} \to (0,1)$ be a differentiable, monotonically increasing latent probability density function, with inverse g^{-1}. We condition on an arbitrary anchor point, ϕ, and then define a map $\varphi_{|\phi}^{[d]} : \{C_{1,s,t}^{[d]}, \ldots, C_{K_t-1,s,t}^{[d]}, S^{[d]}\} \to \{P_{1,s,t}^{[d]}, \ldots, P_{K,s,t}^{[d]}\}$. The induced ordinal probabilities for each of the latent classes are given by,

$$\varphi_{|\phi}^{[d]}(C_{k,s,t}^{[d]}, C_{k-1,s,t}^{[d]}) = g(C_{k,s,t}^{[d]} - \phi) - g(C_{k-1,s,t}^{[d]} - \phi) = P_{k,s,t}^{[d]},\ S^{[d]} = 1 \tag{1.17}$$

with ${\varphi_{|\phi}^{[d]}}^{-1}$ given by,

$${\varphi_{|\phi}^{[d]}}^{-1}(P_{1,s,t}^{[d]}) = g^{-1}(P_{1,s,t}^{[d]}) + \phi = C_{1,s,t}^{[d]} {\varphi_{|\phi}^{[d]}}^{-1}(\mathbf{P_{k,s,t}^{[d]}} | C_{k-1,s,t}^{[d]}) \quad = \phi + g^{-1}\left(P_{k,s,t}^{[d]} + g[C_{k-1,s,t}^{[d]} - \phi] \right) = C_{k,s,t}^{[d]} \tag{1.18}$$

The probability density function for the induced Dirichlet model is given by,

$$\text{Induced-Dir}\left(\mathbf{C}_{s,t}^{[d]}, \mid \boldsymbol{\alpha}_t^{[d]}, \phi \right) = \text{Dir}\left(\mathbf{P}(\mathbf{C}_{s,t}^{[d]}, \phi) \mid \boldsymbol{\alpha}_t^{[d]} \right) \cdot \left| J\left(\mathbf{C}_{s,t}^{[d]} \right) \right|, \tag{1.19}$$

Where $\mathbf{C}_{s,t}^{[d]} = \left(C_{1,s,t}^{[d]}, \ldots, C_{K_t-1,s,t}^{[d]} \right)'$, $\boldsymbol{\alpha}_t^{[d]} = \left(\alpha_{1,t}^{[d]}, \ldots, \alpha_{K_t,t}^{[d]} \right)'$ and $J\left(\mathbf{C}_{s,t}^{[d]} \right)$ is the Jacobian matrix of partial derivatives,

$$J_{k,1}^{[d]} = \frac{\partial P_k^{[d]}}{\partial S^{[d]}} = 1,\ J_{k,k}^{[d]} = \frac{\partial P_k^{[d]}}{\partial C_{k-1}^{[d]}} = -g'\left(C_{k-1}^{[d]} \right),\ J_{k-1,k}^{[d]} = \frac{\partial P_{k-1}^{[d]}}{\partial C_{k-1}^{[d]}} = g'\left(C_{k-1}^{[d]} \right),$$

and zeros everywhere else. We can use the induced Dirichlet model to directly specify a partial pooling model for the Dirichlet parameters $\boldsymbol{\alpha}_t^{[d]}$, so that,

$$\pi\left(\mathbf{C}_{s,t}^{[d]} | \theta \right) = \text{induced-Dir}\left(\mathbf{C}_{s,t}^{[d]} | \boldsymbol{\alpha}_t^{[d]} \right) \cdot \pi\left(\boldsymbol{\alpha}_t^{[d]} \right) \tag{1.20}$$

Where $\mathbf{C}_{s,t}^{[d]} = (C_{1,s,t}^{[d]}, \ldots, C_{K_t-1,s,t}^{[d]})'$, and $\boldsymbol{\alpha}_t^{[d]} = \left(\alpha_{1,t}^{[d]}, \ldots, \alpha_{K_t,t}^{[d]} \right)'$.

In this paper, we use a normal probability density function so that $g(\cdot) = \Phi(\cdot)$ and define the arbitrary anchor point at zero, $\phi = 0$. For prior modelling on the Dirichlet population parameters, we can use a half normal prior $\pi(\boldsymbol{\alpha}_t^{[d]}) = \text{N}_{\geq 0}(\mathbf{a}^{[d]}, \mathbf{b}^{[d]})$ s.t. $\mathbf{a}^{[d]}, \mathbf{b}^{[d]} \in \mathbb{R}_+^{K_t}$ or exponential prior, $\pi(\boldsymbol{\alpha}_t^{[d]}) = \text{exponential}(\mathbf{a}^{[d]})$ s.t. $\mathbf{a}^{[d]} \in \mathbb{R}_+^{K_t}$.

We can order the cutpoint parameters for each study, $C^{[d]}_{k,s,t}$, $k \in \{1, \ldots, K_t - 1\}$ by reparameterizing the cutpoints. We define a map $C^{[d]}_{k,s,t} \mapsto \omega^{[d]}_{k,s,t}$ such that,

$$\omega_{k,s,t} = \begin{cases} C^{[d]}_{1,s,t} \text{if } k = 1, \\ \log\left(C^{[d]}_{k,s,t} - C^{[d]}_{k-1,s,t}\right) \text{if } 1 < k \leq K. \end{cases}$$

Then, to ensure $C^{[d]}_{k,s,t} < C^{[d]}_{k+1,s,t}$, each $C^{[d]}_{k,s,t}$ can be expressed as,

$$C^{[d]}_{k,s,t} = \omega^{[d]}_{1,s,t} + \sum_{i=2}^{k} \exp(\omega^{[d]}_{i,s,t})$$

1.2.3 Modelling conditional dependence between tests and joint test accuracy

We can model the within-study correlation matrices, $\mathbf{\Psi}^{[d]}_s$, using a no pooling model so that $\pi(\mathbf{\Psi}^{[d]}_1, \ldots, \mathbf{\Psi}^{[d]}_S) = \prod_{s=1}^{S} \pi(\mathbf{\Psi}^{[d]}_s)$. We can also use a partial pooling model; since a convex combination of correlation matrices is also a correlation matrix, as suggested by Goodrich[7], the study level correlation matrices can be specified as a weighted linear combination of a summary correlation matrix across studies, $\mathbf{\Psi}^{[d]}_G$,and a matrix of study-level deviations from this $\mathbf{\Psi}^{[d]^\Delta}_s$, with weight $\beta^{[d]}$,

$$\mathbf{\Psi}^{[d]}_s = \left(1 - \beta^{[d]}\right) \cdot \mathbf{\Psi}^{[d]}_G + \beta^{[d]} \cdot \mathbf{\Psi}^{[d]^\Delta}_s, \ \beta^{[d]} \in [0, 1] \tag{1.21}$$

where $\mathbf{\Psi}^{[d]}_G$ is the summary (i.e. global - hence the G subscript) correlation matrix across studies, and $\mathbf{\Psi}^{[d]^\Delta}_s$ is the deviation from $\mathbf{\Psi}^{[d]}_G$ in each study. $\eta_1, \eta_2 \in \mathbb{R}_+$ are constants and $\pi(\beta) = \text{Beta}(a, b)$ s.t. $a, b \in \mathbb{R}_+$. In this case, the population posterior predictive distribution is given by,

$$\mathbf{Z}^{[d]}_G \sim \text{MVN}\left(\boldsymbol{\mu}^{[d]}, \mathbf{\Psi}^{[d]}_G\right), \tag{1.22}$$

Now we discretize $\mathbf{Z}^{[d]}_G$ at a given cutpoint k. Let,

$$y^{[d]}_{G,t,k} = \begin{cases} 0 & \text{if } Z^{[d]}_{G,t} \leq k \\ 1 & \text{if } Z^{[d]}_{G,t} > k \end{cases}$$

We simulate from (1.22) repeatedly and hence obtain ordinal data vectors $\mathbf{y}^{[d]}_{G,t,k}$. Then, we can obtain a summary estimate of Pearson's correlation coefficient between tests t and t' at cutpoints of k and k', within each disease class, $\rho^{[d]}_{G_{tt',kk'}} = \text{Corr}(\mathbf{y}^{[d]}_{G,t,k}, \mathbf{y}^{[d]}_{G,t',k'})$, where Corr denotes the formula for Pearson's correlation coefficient. The summary covariances between tests t and t' at cutpoints of k and k' within each disease class d are given by,

$$\begin{aligned} cov^{[d]}_{G,tt',kk'} &= \rho^{[d]}_{G,tt',kk'} \sqrt{Se_{G,t,k} Se_{G,t',k}(1 - Se_{G,t,k})(1 - Se_{G,t',k'})} \\ cov^{[d]}_{G,tt',kk'} &= \rho^{[d]}_{G,tt',kk'} \sqrt{Sp_{G,t,k} Sp_{G,t',k'}(1 - Sp_{G,t,k}) * (1 - Sp_{G,t',k'})} \end{aligned} \tag{1.23}$$

We can model the conditional dependence between only certain pairs of tests by setting the relevant correlations in $\mathbf{\Psi}^{[d]}_s$ to zero. For the partial pooling model (see equation (1.21)), this can be achieved by setting the relevant terms in $\mathbf{\Psi}^{[d]}_G$ and $\mathbf{\Psi}^{[d]^\Delta}_s$ to zero.

1.2.4 Summary estimates of test accuracy

For dichotomous tests, the summary sensitivity and specificity estimates for test t are given by evaluating equation (1.7) at the means of the partial pooling model (see 1.12),

$$\begin{aligned} Se_{G,t} &= \Phi(\mu^{[1]}_t) \\ Sp_{G,t} &= 1 - \Phi(\mu^{[0]}_t) \end{aligned} \tag{1.24}$$

For ordinal tests, the summary measures of test accuracy for test t at a cutpoint of k are given by equation (1.10) evaluated at the means of the partial pooling model (see 1.12), and, if using a partial pooling model on the cutpoints, at the 'average' cutpoints from the induced Dirichlet partial pooling model (see equation 1.16),

$$\begin{aligned} Se_{G,t,k} &= 1 - \Phi\left(C_{k,t}^{[1]} - \mu_t^{[1]}\right) \\ Sp_{G,t,k} &= \Phi\left(C_{k,t}^{[0]} - \mu_t^{[0]}\right) \end{aligned} \tag{1.25}$$

The summary joint test accuracy for tests t and t' at cutpoints of k and k' are given by,

$$\begin{aligned} Se_{G,tt',kk'}^{BTN} &= Se_{G,t,k} * Se_{G,t',k'} + cov_{G,tt',kk'}^{[1]} \\ Sp_{G,tt',kk'}^{BTN} &= 1 - ((1 - Sp_{G,t,k}) * (1 - Sp_{G,t',k'}) + cov_{G,tt',kk'}^{[0]}) \\ Se_{G,tt',kk'}^{BTP} &= 1 - ((1 - Se_{G,t,k}) * (1 - Se_{G,t',k'}) + cov_{G,tt',kk'}^{[1]}) \\ Sp_{G,tt',kk'}^{BTP} &= Sp_{G,t,k} * Sp_{G,t',k'} + cov_{G,tt',kk'}^{[0]} \end{aligned} \tag{1.26}$$

Where BTP and BTN are 'believe the positives' and 'believe the negatives' testing strategies, respectively. The former refers to a testing strategy where – as the name implies – all patients undertake a test, then only that subset of patients who test positive on this test are referred to undertake a second test. Believe the negatives (BTN) is the opposite – only patients who test negative on the first test go on to receive the second test.

We can generate predictions for a 'new' $(S+1)$-th study by simulating a draw (at each iteration) from the posterior predictive distributions of the between-study normal hierarchical model, (see (1.12)), $\boldsymbol{\nu}_{S+1,t}$, and, if using a partial pooling model on the cutpoints, a new vector of cutpoints from the induced Dirichlet cutpoint model (see (1.16)), $\mathbf{C}_{S+1,t}^{[d]}$. The predicted sensitivities and specificities for an $(S+1)$-th study are given by $Se_{S+1,t} = \Phi(\nu_{S+1,t}^{[1]})$ and $Sp_{S+1,t} = 1 - \Phi(\nu_{S+1,t}^{[0]})$ for dichotomous tests, and $Se_{S+1,t,k} = 1 - \Phi\left(C_{S+1,k,t}^{[1]} - \nu_{S+1,t}^{[1]}\right)$ and $Sp_{S+1,t,k} = \Phi\left(C_{S+1,k,t}^{[0]} - \nu_{S+1,t}^{[0]}\right)$ for ordinal tests.

1.3 Posterior predictive checking and model comparison

For posterior predictive checks, we can re-construct the study-specific 2x2 tables between tests t and t' by dichotomising the tests at a given cutpoint. We calculate the probability of observing each test pattern by applying the TLCM formulae[8,9,10],

$$\begin{aligned} Pr(+_{tk}, +_{t'k'})_s &= p_s * (Se_{s,t,k} * Se_{s,t',k'} + cov_{s,tt',kk'}^{[1]}) + (1-p_s) * ((1 - Sp_{s,t,k}) * (1 - Sp_{s,t',k'}) + cov_{s,tt',kk'}^{[0]}) \\ Pr(+_{tk}, -_{t'k'})_s &= p_s * (Se_{s,t,k} * (1 - Se_{s,t',k'}) - cov_{s,tt',kk'}^{[1]}) + (1-p_s) * ((1 - Sp_{s,t,k}) * (Sp_{s,t',k'}) - cov_{s,tt',kk'}^{[0]}) \\ Pr(-_{tk}, +_{t'k'})_s &= p_s * ((1 - Se_{s,t,k}) * Se_{s,t',k'} - cov_{s,tt',kk'}^{[1]}) + (1-p_s) * (Sp_{s,t,k} * (1 - Sp_{s,t',k'}) - cov_{s,tt',kk'}^{[0]}) \\ Pr(-_{tk}, -_{t'k'})_s &= p_s * ((1 - Se_{s,t,k}) * (1 - Se_{s,t',k'}) + cov_{s,tt',kk'}^{[1]}) + (1-p_s) * ((1 - Sp_{s,t,k}) * (1 - Sp_{s,t',k'}) + cov_{s,tt',kk'}^{[0]}) \end{aligned} \tag{1.27}$$

Where $cov_{s,tt',kk'}^{[d]}$ represent the study-specific covariances between tests t and t' at cutpoints k and k'. Then, we can calculate the model-predicted cell counts by multiplying each probability in (1.27) by the number of individuals in each study, N_s. We can plot the model-predicted 2x2 tables against the observed 2x2 tables to inspect the fit. We can also plot the model-predicted correlations against the observed correlations to assess model fit, using the correlation residual plot proposed by Qu et al[11]. The model-predicted correlations are given by,

$$\begin{aligned} \rho_{tk,t'k'} &= \frac{Pr(+_{tk}, +_{t'k'})_s - Pr(+_{tk})Pr(+_{t'k'})}{\sqrt{Pr(+_{tk})Pr(+_{t'k'})(1 - Pr(+_{tk}))(1 - Pr(+_{t'k'}))}}, \text{where} \\ Pr(+_{tk}) &= Pr(+_{tk}, +_{t'k'})_s + Pr(+_{tk}, -_{t'k'})_s \\ Pr(+_{t'k'}) &= Pr(+_{tk}, +_{t'k'})_s + Pr(-_{tk}, +_{t'k'})_s \end{aligned} \tag{1.28}$$

For model comparison, we can conduct estimated leave-one-out (LOO) cross-validation[12]. We used the 'loo' package[13], which computes the estimated LOO statistic using Pareto-smoothed importance

sampling (PSIS-LOO). LOO is superior to both the deviance information criterion (DIC) and the widely applicable information criterion (WAIC). This is because the DIC is not fully Bayesian, as it is based on a point estimate[14], and the LOO is invariant to parametrisation. Furthermore, both the DIC and the WAIC lack diagnostics, and the LOO is also more robust than both the DIC and the WAIC in the face of weak priors or influential observations[12].

2 Detailed description of prior model used for case study

For the imperfect gold standard (ultrasound), we incorporated subject-matter knowledge used informative priors based on the available literature. More specifically, a systematic review and meta-analysis[15] estimated the sensitivity of ultrasound to be 0.94 (95% confidence interval [ConfI] = [0.93, 0.95]) and 0.64 (95% ConfI = [0.60, 0.67]) for proximal and distal DVT, respectively. It estimated the specificity for either type of DVT to be 0.94 (95% ConfI = [0.93, 0.94]). Another systematic review investigating the accuracy of various tests specifically for proximal DVT[16] found that sensitivity varied from 0.84 (95% ConfI = [0.72, 0.97]) to 0.97 (95% ConfI = [0.90, 1.00]) and the specificity varied from 0.93 (95% ConfI = [0.80, 1.00]) to 0.96 (95% ConfI = [0.87, 1.00]) for ultrasound. As a result of this, we used an informative $\mu_1^{[1]} \sim N(0.75, 0.40)$ prior, which corresponds to a 95% prior interval of $(0.49, 0.94)$ for the sensitivity, and $\mu_1^{[0]} \sim N(-1.70, 0.40)$ which corresponds to a 95% prior interval of $(0.82, 0.99)$ for the specificity.

We used $N(0, 1)$ priors for the means of the sensitivities and specificities of the D-Dimer and Well's score on the probit scale i.e. $\mu_t^{[d]} \sim N(0, 1)$ s.t. $t \in \{2, 3\}$, which correspond to flat priors on the probability scale. We fit all models using weak $N_{\geq 0}(0, 0.5)$ priors for the between-study standard deviations, so that $\sigma_t^{[d]} \sim N_{\geq 0}(0, 0.5)$ s.t. $t \in \{1 = \text{Reference}, 2 = \text{D-Dimer}, 3 = \text{Wells}\}$, $d \in \{0, 1\}$. These are weak priors since they weakly pull the study-specific sensitivities and specificities towards each other, whilst allowing a large between-study variation in accuracy estimates if the data demands. This is because a shift of 0.5 on the probit scale represents a large change on the sensitivity or specificity estimate. For example, if 0.8 is the value found for the summary sensitivity, and if $\sigma = 1$, then we would expect the study-specific estimates would be in the range of $(0.63, 0.91)$ with a 95% probability and if $\sigma = 2$ then they would be in the range of $(0.44, 0.97)$ with 95% probability. We also used weak priors on the between-study correlation parameters (see equation 1.12), so that $\mathbf{\Omega}_t \sim \text{LKJcorr}(2)\ \forall t$. For the conditional dependence models, we used the partial pooling model on the within-study correlations (see equation 1.21), with prior model $\beta \sim Beta(2, 2)$, $\mathbf{\Omega}_G^{[d]} \sim \text{LKJcorr}(4)$ and $\mathbf{\Omega}_s^{[d]\Delta} \sim \text{LKJcorr}(4)$.

3 Prior and posterior predictive checks for case study analysis

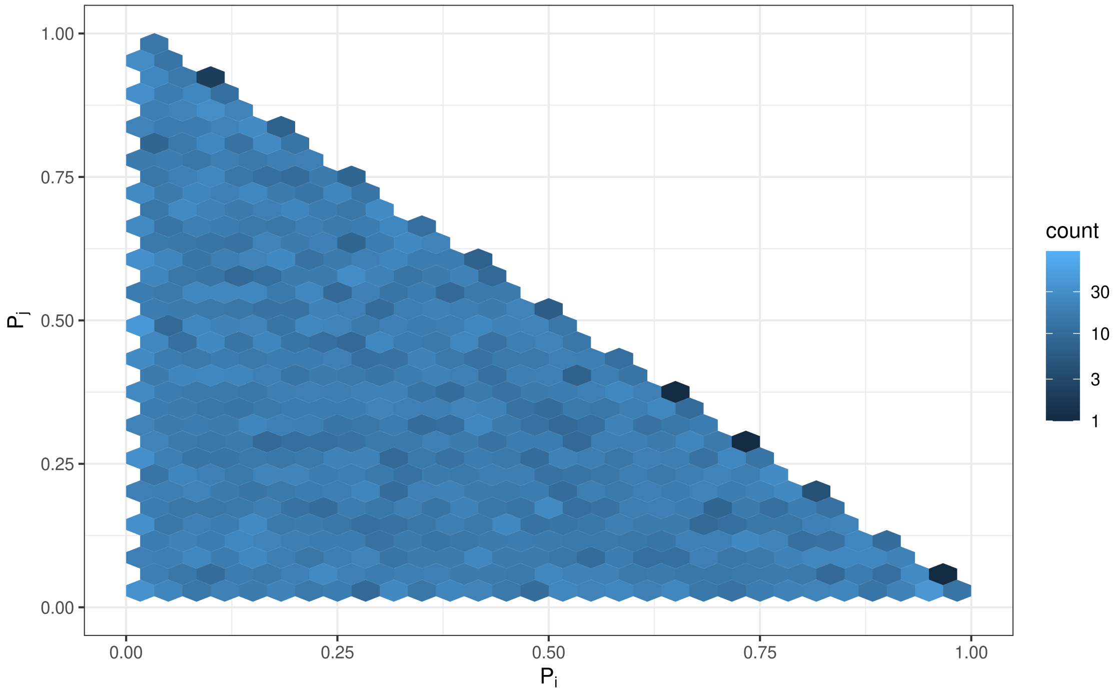

Figure 1: Prior predictive check for $\kappa^{[d]} \sim N_{\geq 0}(0, 50)$ prior. Note that the count is out of a total of 10,000 simulations

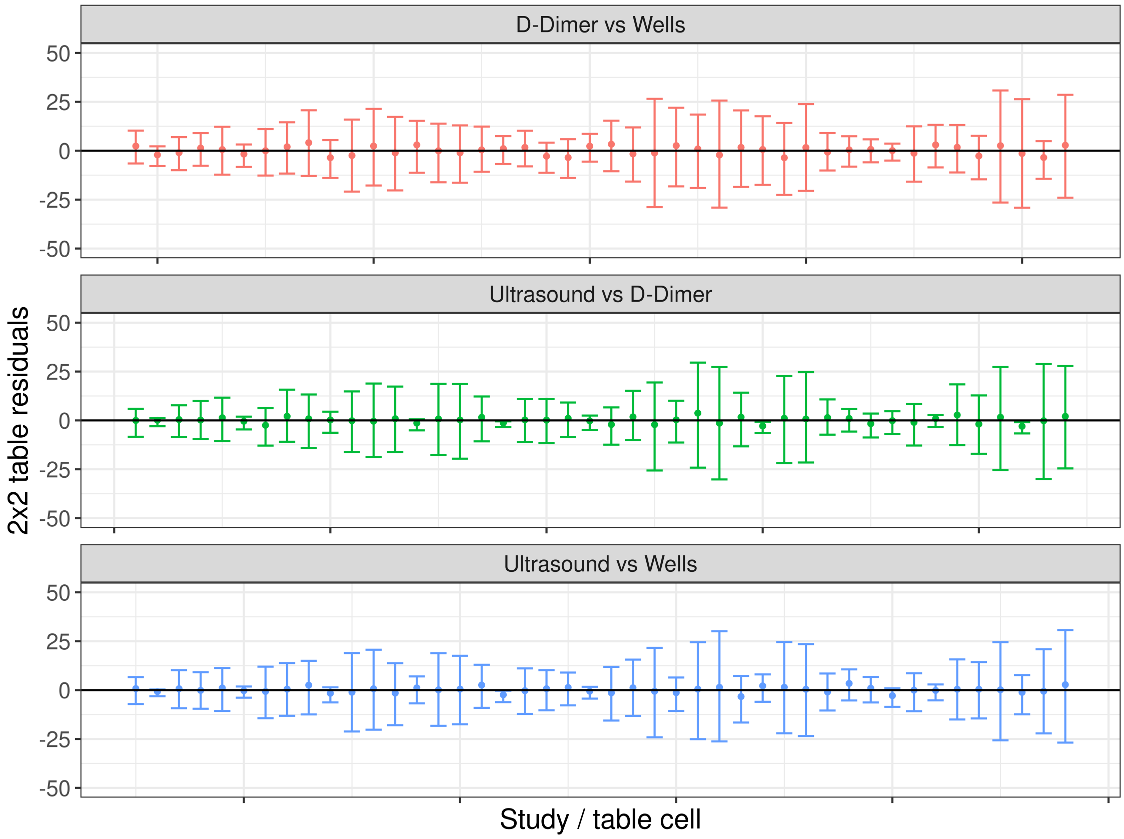

Figure 2: Posterior predictive check for model 4; 2x2 table count residual plot

4 Induced Dirichlet cutpoint model (Betancourt, 2019)

The induced Dirichlet model allows us to move away from the abstract latent space in which the cutpoints are defined, and applies a Dirichlet model directly to the ordinal probabilities. We need to find an injective (i.e. one-to-one) function which maps the latent cut point parameters in each study $\{C^{[d]}_{1,s,t}, \ldots, C^{[d]}_{K_t-1,s,t}\}$ to the ordinal probabilities $\{P^{[d]}_{1,s,t}, \ldots, P^{[d]}_{K_t,s,t}\}$. Let $S^{[d]} = \sum_{k=1}^{K} P^{[d]}_{k,s,t} = 1$ and let $g : \mathbb{R} \to (0, 1)$ be a differentiable, monotonically increasing latent probability density function, with inverse g^{-1}. We condition on an arbitrary anchor point, ϕ, and then define a map $\varphi^{[d]}_{|\phi} : \{C^{[d]}_{1,s,t}, \ldots, C^{[d]}_{K_t-1,s,t}, S^{[d]}\} \to \{P^{[d]}_{1,s,t}, \ldots, P^{[d]}_{K,s,t}\}$. The induced ordinal probabilities for each of the latent classes are given by,

$$\varphi^{[d]}_{|\phi}(C^{[d]}_{k,s,t}, C^{[d]}_{k-1,s,t}) = g(C^{[d]}_{k,s,t} - \phi) - g(C^{[d]}_{k-1,s,t} - \phi) = P^{[d]}_{k,s,t},\ S^{[d]} = 1 \tag{4.1}$$

with ${\varphi^{[d]}_{|\phi}}^{-1}$ given by,

$$\begin{aligned} {\varphi^{[d]}_{|\phi}}^{-1}(P^{[d]}_{1,s,t}) &= g^{-1}(P^{[d]}_{1,s,t}) + \phi = C^{[d]}_{1,s,t} \\ {\varphi^{[d]}_{|\phi}}^{-1}(\mathbf{P^{[d]}_{k,s,t}} | C^{[d]}_{k-1,s,t}) &= \phi + g^{-1}\left(P^{[d]}_{k,s,t} + g[C^{[d]}_{k-1,s,t} - \phi]\right) = C^{[d]}_{k,s,t} \end{aligned} \tag{4.2}$$

The probability density function for the induced Dirichlet model is given by,

$$\text{Induced-Dir}\left(\mathbf{C}^{[d]}_{s,t}, \mid \boldsymbol{\alpha}^{[d]}_t, \phi\right) = \text{Dir}\left(\mathbf{P}(\mathbf{C}^{[d]}_{s,t}, \phi) \mid \boldsymbol{\alpha}^{[d]}_t\right) \cdot \left| J\left(\mathbf{C}^{[d]}_{s,t}\right)\right|, \tag{4.3}$$

Where $\mathbf{C}^{[d]}_{s,t} = \left(C^{[d]}_{1,s,t}, \ldots, C^{[d]}_{K_t-1,s,t}\right)'$, $\boldsymbol{\alpha}^{[d]}_t = \left(\alpha^{[d]}_{1,t}, \ldots, \alpha^{[d]}_{K_t,t}\right)'$ and $J\left(\mathbf{C}^{[d]}_{s,t}\right)$ is the Jacobian matrix of partial derivatives,

$$J^{[d]}_{k,1} = \frac{\partial P^{[d]}_k}{\partial S^{[d]}} = 1,\ J^{[d]}_{k,k} = \frac{\partial P^{[d]}_k}{\partial C^{[d]}_{k-1}} = -g'\left(C^{[d]}_{k-1}\right),\ J^{[d]}_{k-1,k} = \frac{\partial P^{[d]}_{k-1}}{\partial C^{[d]}_{k-1}} = g'\left(C^{[d]}_{k-1}\right),$$

and zeros everywhere else. We can use the induced Dirichlet model to directly specify a partial pooling model for the Dirichlet parameters $\boldsymbol{\alpha}^{[d]}_t$, so that,

$$\pi\left(\mathbf{C}^{[d]}_{s,t} | \theta\right) = \text{induced-Dir}\left(\mathbf{C}^{[d]}_{s,t} | \boldsymbol{\alpha}^{[d]}_t\right) \cdot \pi\left(\boldsymbol{\alpha}^{[d]}_t\right) \tag{4.4}$$

Where $\mathbf{C}^{[d]}_{s,t} = (C^{[d]}_{1,s,t}, \ldots, C^{[d]}_{K_t-1,s,t})'$, and $\boldsymbol{\alpha}^{[d]}_t = \left(\alpha^{[d]}_{1,t}, \ldots, \alpha^{[d]}_{K_t,t}\right)'$.

In this paper, we use a normal probability density function so that $g(\cdot) = \Phi(\cdot)$ and define the arbitrary anchor point at zero, $\phi = 0$. For prior modelling on the Dirichlet population parameters, we can use a half normal prior $\pi(\boldsymbol{\alpha}^{[d]}_t) = \text{N}_{\geq 0}(\mathbf{a}^{[d]}, \mathbf{b}^{[d]})$ s.t. $\mathbf{a}^{[d]}, \mathbf{b}^{[d]} \in \mathbb{R}^{K_t}_+$ or exponential prior, $\pi(\boldsymbol{\alpha}^{[d]}_t) = \text{exponential}(\mathbf{a}^{[d]})$ s.t. $\mathbf{a}^{[d]} \in \mathbb{R}^{K_t}_+$.

We can order the cutpoint parameters for each study, $C^{[d]}_{k,s,t}$, $k \in \{1, \ldots, K_t - 1\}$ by reparameterizing the cutpoints. We define a map $C^{[d]}_{k,s,t} \mapsto \omega^{[d]}_{k,s,t}$ such that,

$$\omega_{k,s,t} = \begin{cases} C^{[d]}_{1,s,t} & \text{if } k = 1, \\ \log\left(C^{[d]}_{k,s,t} - C^{[d]}_{k-1,s,t}\right) & \text{if } 1 < k \leq K. \end{cases}$$

Then, to ensure $C^{[d]}_{k,s,t} < C^{[d]}_{k+1,s,t}$, each $C^{[d]}_{k,s,t}$ can be expressed as,

$$C^{[d]}_{k,s,t} = \omega^{[d]}_{1,s,t} + \sum_{i=2}^{k} \exp(\omega^{[d]}_{i,s,t})$$

5 Generating the truncated multivariate normal densities (Geweke, Hajivassiliou and Keane [1994] algorithm and Goodrich [2017])

Implementing the likelihood for each study requires integrating over truncated multivariate normal densities. We did this in Stan by using the method from Goodrich 2017[17], which uses the GHK algorithm[18]. We will summarise the method described in Goodrich 2017[17] below.

We can parametrise the multivariate normal densities to be truncated for each study in terms of its Cholesky factor. We notional simplicity denote $\mathbf{z} = \mathbf{Z}_{s,n}$, $\boldsymbol{\nu} = \boldsymbol{\nu}_s{}^{[d]}$, and let $\boldsymbol{\Psi} = \boldsymbol{\Psi}_s^{[d]}$. We can write each multivariate normal distribution, $\mathbf{z}$, as

$$\mathbf{z} = \ ^\circ + \mathbf{L} \cdot \mathbf{x}$$

Where $x \sim N(0,1)$ and L is the Cholesky factor matrix of $\boldsymbol{\Psi} = \mathbf{L} \cdot \mathbf{L}^T$.

We can write this as,

$$\begin{bmatrix} \mathbf{x}_1 \\ x_k \\ \mathbf{x}_3 \end{bmatrix} = \begin{bmatrix} \boldsymbol{\nu}_1 \\ \nu_k \\ \boldsymbol{\nu}_3 \end{bmatrix} + \begin{bmatrix} \mathbf{L}_{11} & \mathbf{0} & \mathbf{0} \\ \mathbf{L}_{k1} & L_{kk} & \mathbf{0} \\ \mathbf{L}_{31} & \mathbf{L}_{3k} & \mathbf{L}_{33} \end{bmatrix} \cdot \begin{bmatrix} \mathbf{z}_1 \\ z_k \\ \mathbf{z}_3 \end{bmatrix}$$

Where $\mathbf{L}_{11}$ and $\mathbf{L}_{33}$ are lower triangular submatrices, $\mathbf{L}_{31}$ is a submatrix, $L_{kk} \in \mathbb{R}_+$ is a scalar, $\mathbf{L}_{k1} \in \mathbb{R}^{1\times(k-1)}$ contains the elements of $\mathbf{L}$ to the left of L_{kk}, and $\mathbf{L}_{3k} \in \mathbb{R}^{k-1}$ contains the elements below L_{kk}.

Let $x(u) = \Phi^{-1}(u)$ where $u \sim \text{Uniform}(0,1)$, i.e. $x(u)$ can be generated by the inverse CDF method, so that we can write $\mathbf{z}$ as

$$\mathbf{z} = \ ^\circ + \mathbf{L} \cdot \mathbf{x}(\mathbf{u})$$

Suppose that we have a bound, B_1, on the first element of $z_1 = \nu_1 + L_{11} \cdot x(u_1)$. Then, the constraint binds at $x^*(u_1) = \frac{B_1 - \nu_1}{L_{11}}$, and $u_1^* = \Phi\left(\frac{B_1-\nu_1}{L_{11}}\right)$. If $B_1 = \overline{B_1}$ is an upper bound on z_1 then $v_1 = u_1 \cdot u_1^* \sim \text{Uniform}(0, u_1^*)$ since $u_1 \sim \text{Uniform}(0,1)$, with $\pi(v_1) = \frac{1}{u_1^*}$. If $B_1 = \overline{B_1}$ is a lower bound on z_1 then $v_1 = u_1^* + (1-u_1^*) \cdot u_1 \sim \text{Uniform}(u_1^*, 1)$ with $\pi(v_1) = \frac{1}{1-u_1^*}$. If we have both an upper and lower bound, then $v_1 = \underline{u_1^*} + (\overline{u_1^*} - \underline{u_1^*}) \cdot u_1 \sim \text{Uniform}(\underline{u_1^*}, \overline{u_1^*})$ with $\pi(v_1) = \frac{1}{\overline{u_1^*} - \underline{u_1^*}}$. Then, given u_1 we can consider a known bound, B_2 , on the second element $z_2 = \nu_2 + L_{21} \cdot x_1 + L_{22} \cdot x(u_2)$ of $\mathbf{z}$. Following the same steps as before, we solve for $u_2^* = \Phi\left(\frac{B_2 - (\nu_2 + L_{21} \cdot x_1)}{L_{22}}\right)$, with $\pi(v_2) = \frac{1}{u_2^*}$ if $B_2 = \underline{B_2}$, $\pi(v_2) = \frac{1}{1-u_2^*}$ if $B_2 = \overline{B_2}$, and $\pi(v_2) = \frac{1}{\overline{u_2^*} - \underline{u_2^*}}$ if we have both an upper and lower bound.

In general, given $\mathbf{x}_1 = \Phi^{-1}(\{u_1, \ldots, u_{k-1}\})$ we can consider a known bound B_k on $z_k = \nu_k + \mathbf{L}_{k1} \cdot x_1 + L_{kk} \cdot x_k$ and solve for $u_k^* = \Phi\left(\frac{B_k - (\nu_k + \mathbf{L}_{k1} \cdot x_1)}{L_{kk}}\right)$. Then,

$$\pi(v_k | u_k^*) = \begin{cases} \frac{1}{u_k^*} & \text{if we have an upper bound} \\ \frac{1}{1-u_k^*} & \text{if we have a lower bound} \\ \frac{1}{\overline{u_k^*} - \underline{u_k^*}} & \text{if we have both an upper and lower bound} \end{cases}$$

Stan only allows bounds on vectors declared in the parameters block, so we need to declare the u_k as nuisance parameters, and construct each v_k. Since v_k is a transformed parameter, we need a Jacobian adjustment, i.e. we need to adjust the log-kernel by the log of the absolute value of the derivative of the transformation function $v_k \to u_k$,

$$\log\left(\left|\frac{\mathrm{d}v_k}{\mathrm{d}u_k}\right|\right) = \begin{cases} \log(u_k^*) & \text{if we have an upper bound} \\ \log(1 - u_k^*) & \text{if we have a lower bound} \\ \log(\overline{u_k^*} - \underline{u_k^*}) & \text{if we have both an upper and lower bound} \end{cases}$$

6 Estimated number of parameters & identifiability

In this section, we will derive general equations for the number of parameters that our proposed MVP-LC model uses. Since our proposed MVP-LC model is Bayesian, our derivation here assumes improper prior distributions - that is, the priors for all parameters can take on any value within its defined range (e.g., between 0 and ∞ for standard deviation parameters). Let S denote the number of studies in the meta-analysis, and let $T = T_d + T_o$ denote the numbers of tests, where T_d and T_o are the number of dichotomous and ordinal tests in each study $s \in \{1, ...S\}$, respectively. We will refer to a given ordinal test as $t = t_o$ ($t_o \in \{1, ..., T_o\}$) and dichotomous tests as $t = t_d$ ($t_d \in \{1, ..., T_d\}$) Let K_t denote the number of categories for some ordinal test $t = t_o$ ($t \in \{1, ..., T_o\}$).

For both ordinal and dichotomous tests, our MVP-LC model will need to estimate the following parameters, which are shared between studies - those for the bivariate between-study partial pooling model: $2 \cdot T$ summary-level means ($\mu_t^{[d]}$); $2 \cdot T$ between-study standard deviations ($\sigma_t^{[d]}$); and T between-study correlations (ρ_t). For the within-study correlation partial pooling model we have 2 weights ($\beta^{[d]}$) and $2 \cdot \binom{T}{2} = T \cdot (T-1)$ study-level polychoric correlation parameters ($\epsilon_{s,t,t'}^{[d]}$).

Each ordinal test $t = t_o$ requires an additional $2 \cdot K_t - 2$ parameters to be estimated, due to the summary cutpoint parameters $\left(C_{1,t}^{[d]}, \ldots, C_{K_t-1,t}^{[d]}\right)'$ in the Induced Dirichlet partial pooling cutpoint model[6] (see section 4), one of which does not need to be estimated as it is arbitrary. We can now see that dichotomous tests require $5 \cdot T_d + T_d \cdot (T_d - 1) + 2 = 4 \cdot T_d + T_d^2 + 2$ shared parameters to be estimated, and ordinal tests require a total of $\sum_{t=1}^{T_o} 2 \cdot (K_{t_o} - 2) + \left[4 \cdot T_o + T_o^2 + 2\right]$ between-study parameters to be estimated.

For both ordinal and dichotomous tests, our MVP-LC model will need to estimate the following study-specific parameters - up to $2 \cdot T \cdot S$ study-specific means ($\nu_{s,t}^{[d]}$), which are drawn from the between-study bivariate partial pooling model. More specifically, the number of parameters are $2 \cdot T \cdot X$ with $0 < X < S$ - with X being closer to 0 if there is little between-study heterogeneity present, or closer to S if there is extreme heterogeneity. For the partial pooling model for the within-study correlations ($\epsilon_{s,t,t'}^{[d]}$, the elements of $\mathbf{\Psi}_s^{[d]}$), we need to estimate up to $X \cdot T \cdot (T-1)$ study-level polychoric correlation parameters ($0 < X < S$). For the no pooling prevalence model, we have to estimate S prevalence parameters (p_s) in each study. Ordinal tests require up to an additional $2 \cdot (K_t - 2) \cdot X$ (where $0 < X < S$) parameters to be estimated, due to the cutpoints parameters $(C_{1,t}^{[d]}, \ldots, C_{K_t-1,t}^{[d]})'$, one of which does not need to be estimated as it is arbitrary.

We can now see that dichotomous tests require $X \cdot T_d + X \cdot T_d^2 + S$ study-specific parameters to be estimated ($0 < X < S$), and ordinal tests require a total of $\sum_{t=1}^{T_o} 2 \cdot (K_{t_o} - 2) \cdot X + \left[X \cdot T_o + X \cdot T_o^2 + S\right]$ study-specific parameters to be estimated. Overall, we need to estimate: $T_d \cdot (4 + X) + T_d^2 \cdot (1 + X) + 2 + S$ parameters for dichotomous tests and $\sum_{t=1}^{T_o} 2 \cdot (K_{t_o} - 2) + \sum_{t=1}^{T_o} 2 \cdot (K_{t_o} - 2) \cdot X + \left[T_o \cdot (4 + X) + T_o^2 \cdot (1 + X) + 2 + S\right]$ parameters for ordinal tests, making a total number of:

$$(T_d + T_o)(4 + X) + (1 + X) \cdot \left[T_d^2 + T_o^2 + 2 \cdot \sum_{t_o=1}^{T_o} K_{t_o} + 4 \cdot (K_{t_o} - T_o)\right] + 2 \cdot S + 4$$

parameters to be estimated. As we mentioned, this does not account for prior distributions.

Dichotomous test accuracy data (i.e. 2x2 tables) contribute 3 degrees of freedom DOFs for each dichotomous test (t_d) for each study s, since all studies evaluate the same tests T. However, ordinal test accuracy data contributes $K_{t_o}^2 - 1$ DOFs for each study and ordinal test t_o, with the DOF:parameter ratio increasing as the number of categories K_{t_o} increases. This can be shown by using the general equations that we have derived above.
